# Supplementary material for: Human genetic evidence to inform clinical development of interleukin-6 signaling inhibition for abdominal aortic aneurysm
Source: Arterioscler Thromb Vasc Biol. Author manuscript; Available in PMC 2025 Feb 24. (PMC7617413; doi:10.1161/ATVBAHA.124.321988)

Human genetic evidence to inform clinical development of interleukin-6 signaling inhibition for abdominal aortic aneurysm

SUPPLEMENTARY MATERIALS

Table S1. Genetic variants included in the extended instrument.

rsid	Chromosome and position (GRCh37)
**rs73026617**	chr1:154369981
**rs12083537**	chr1:154381103
**rs4556348**	chr1:154394296
**rs2228145**	chr1:154426970
**rs11264224**	chr1:154568086
**rs12059682**	chr1:154579585
**rs34693607**	chr1:154661369

Table S2. List of codes used to define any rheumatological disorder and any connective tissue disorder.

	**Codes used**		
**Disorder**	**ICD-9**	**ICD-10**	**Self-reported**
**Rheumatologic disorders**			
**Giant cell arthritis**	446.5	M31.[5,6]	1376
**Takayasu arteritis**	446.7	M31.4	-
**Behçet disease**	136.1	M35.2	-
**Cogan's syndrome**	370.52	H16.3	-
**Anti-neutrophil cytoplasmic antibody-associated vasculitis**	446.4	M31.3	1378
**Ankylosing spondylitis**	720.0	M45.X	1313
**Relapsing polychondritis**		M94.1	
**Sarcoidosis**	135.X	D86.X	1371
**Systemic lupus erythematosus**	710.0	M32.[0,1,8,9]	1424
**Rheumatoid arthritis**	714.[0,1,2,81]	M05.[0-3,8,9], M06.[0,8,9]	1464
**Other non-specific rheumatological disorder codes**	279.49	D89.9	-
**Connective tissue disorders**			
**Marfan syndrome**	759.82	Q87.4	-
**Ehlers-Danlos syndrome**	757.83	Q79.6	-
**Other non-specific connective tissue disorder codes**	-	L94.X	1373

Figure S1. Genetic associations with circulating interleukin-6 and C-reactive protein levels in the neighborhood of the *IL6R* gene region (±100kb).

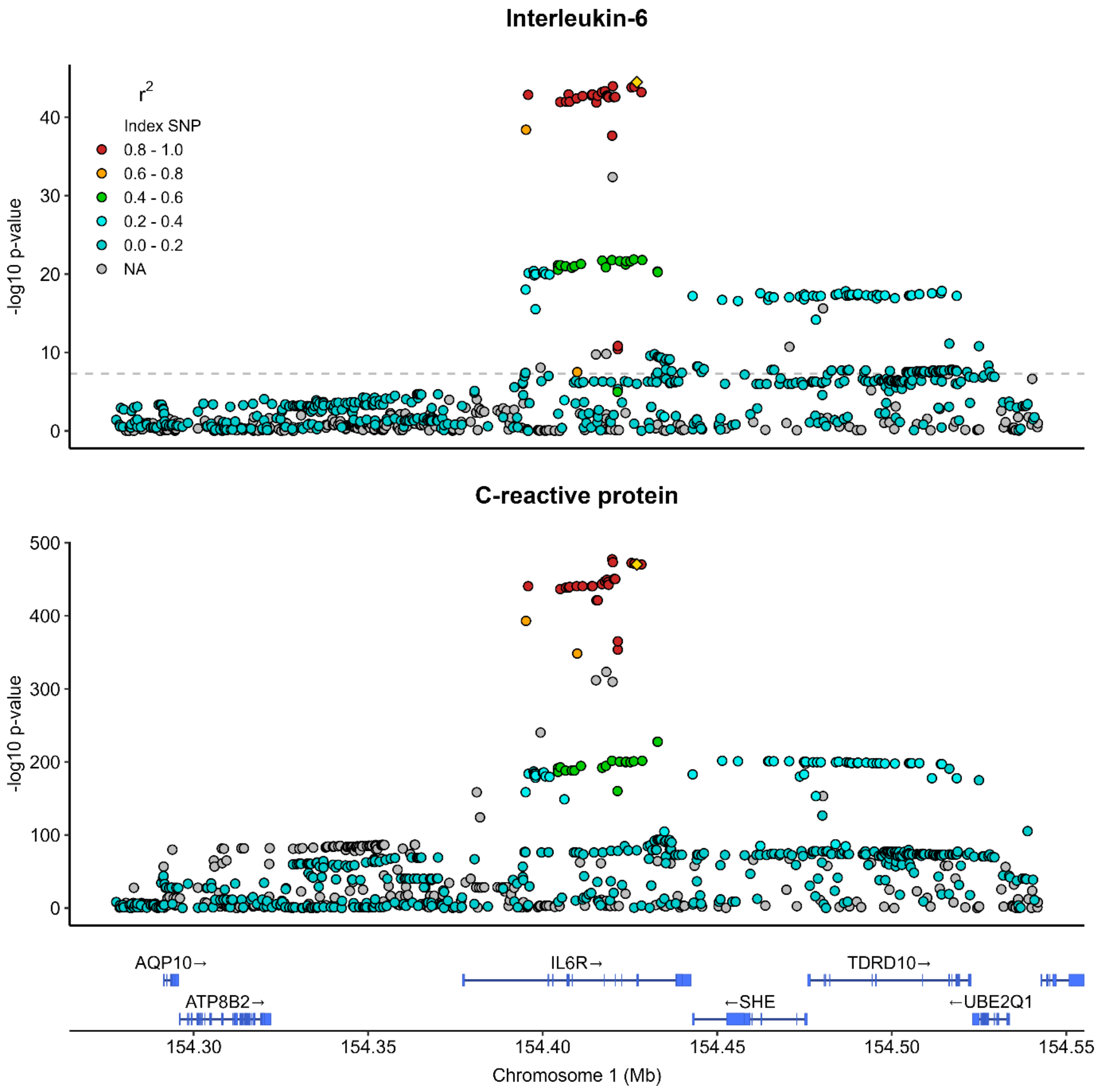

Figure S2. Scatterplot showing associations of genetic variants in the *IL6R* gene region with C-reactive protein and abdominal aortic aneurysm (AAA) risk

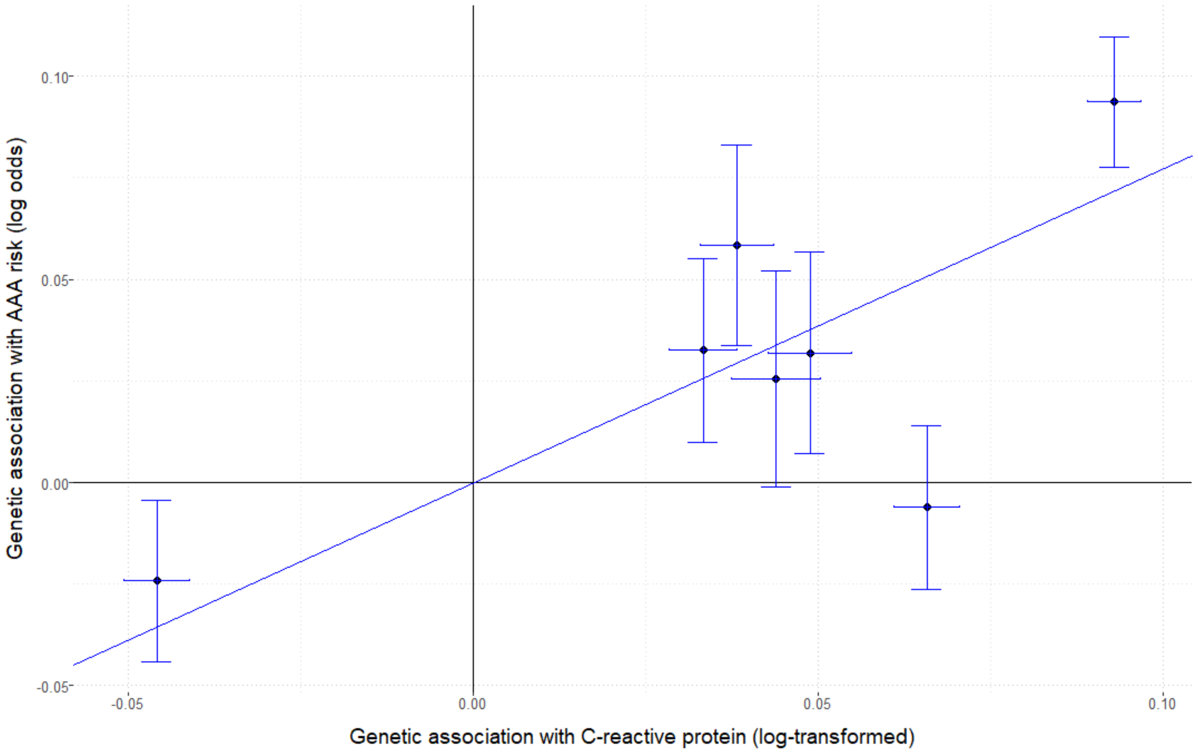

Supplement: Supplementary material [file EMS202956-supplement-Supplementary_material.pdf]
